# Synthesis of Polyurethane Membranes Derived from Red Seaweed Biomass for Ammonia Filtration

**DOI:** 10.3390/membranes11090668

**Published:** 2021-08-30

**Authors:** Salfauqi Nurman, Saiful Saiful, Binawati Ginting, Rahmi Rahmi, Marlina Marlina, Yusuf Wibisono

**Affiliations:** 1 Graduate School of Mathematics and Applied Sciences, Universitas Syiah Kuala, Banda Aceh 23111, Indonesia; salfauqi@mhs.unsyiah.ac.id; 2 Department of Agricultural Industrial Engineering, Faculty of Agricultural Technology, Universitas Serambi Mekkah, Banda Aceh 23245, Indonesia; 3 Politeknik Pelayaran Malahayati, Aceh Besar 23381, Indonesia; 4 Department of Chemistry, Faculty of Mathematics and Natural Sciences, Universitas Syiah Kuala, Banda Aceh 23111, Indonesia; bina_laras@yahoo.com (B.G.); rahmi@fmipa.unsyiah.ac.id (R.R.); marlina@unsyiah.ac.id (M.M.); 5 Department of Bioprocess Engineering, Faculty of Agricultural Technology, Brawijaya University, Malang 65141, Indonesia; Y_Wibisono@ub.ac.id

**Keywords:** ammonia, biomass, *Gracilaria verrucosa* Greville, polyurethane membranes, response surface methodology, toluene diisocyanate

## Abstract

The development of membrane technology is rapidly increasing due to its numerous advantages, including its ease of use, chemical resistant properties, reduced energy consumption, and limited need for chemical additives. Polyurethane membranes (PUM) are a particular type of membrane filter, synthesized using natural organic materials containing hydroxy (-OH) groups, which can be used for water filtration, e.g., ammonia removal. Red seaweed (Rhodophyta) has specific molecules which could be used for PUM. This study aimed to ascertain PUM synthesis from red seaweed biomass (PUM-RSB) by using toluene diisocyanate via the phase inversion method. Red seaweed biomass with a particle size of 777.3 nm was used as starting material containing abundant hydroxy groups visible in the FTIR spectrum. The PUM-RSB produced was elastic, dry, and sturdy. Thermal analysis of the membrane showed that the initial high degradation temperature was 290.71 °C, while the residue from the thermogravimetric analysis (TGA) analysis was 4.88%. The PUM-RSB section indicates the presence of cavities on the inside. The mechanical properties of the PUM-RSB have a stress value of 53.43 MPa and a nominal strain of 2.85%. In order to optimize the PUM-RSB synthesis, a Box–Behnken design of Response Surface Methodology was conducted and showed the value of RSB 0.176 g, TDI 3.000 g, and glycerin 0.200 g, resulting from the theoretical and experimental rejection factor, i.e., 31.3% and 23.9%, respectively.

## 1. Introduction

Membrane technology continues to develop from year to year. As a separation and purification method, membrane technology has several advantages, including minimum energy use, less requirement for additional chemicals, and adequate chemical resistance [1]. The membrane works in extreme pH conditions [2], is very easy to apply [3], is practical, simple [4,5,6], and has many applications [7]. Membranes can be synthesized using inorganic or organic materials. A particular organic membrane that can be synthesized is a polyurethane membrane PUM [8].

PUM can be synthesized by using various natural materials such as castor oil [9], rubber seed oil [10], avocado seed oil [11], nyamplung seed oil [12], and carrageenan [13]. The natural materials, which contain a large amount of hydroxy (-OH) groups, can form urethane bonds with a source of isocyanate (-NCO) [10,14,15]. Selected natural materials that contain many hydroxy groups are red seaweed [8,16] and isolated carrageenan from seaweed [16].

Red seaweed, mainly *Gracilaria verrucosa* Greville, is an abundant and often underutilized natural substance—especially in Indonesia’s Aceh region. The dominant components of red seaweed include carrageenan, alginate, and agar [5,16]. Red seaweed has great potential as a primary material for PUM synthesis, due to its -OH content [5,8,16]. The carrageenan from seaweed has been used as a basic material for the synthesis of PUM with optimum reaction conditions obtained at 60 °C for 5 min with a ratio concentration of carrageenan to toluene diisocyanate of 15% (*w*/*v*). The analysis of test data on carrageenan PUM showed that the properties of the resulting membrane were slightly elastic, where the elongation percentage was only 9%. The membrane also has high tensile strength, 340 kgf/mm^2^, 9% elongation, 243.6 °C glass transition temperature, and a 423.02 °C melting point. The membrane performance is assessed by applying it to the ultrafiltration process using standard 1000 ppm dextran solution. The average flux obtained is 39.2 L/h flux value and a rejection factor of 45.9% [13].

One exciting application of PUM is in ammonia gas sensors [17] and for removing ammonia from the air [18], river water [19], wastewater [20], saline wastewater [21], and NH_4_Cl solutions [5]. Carrageenan contained in red seaweed has the anion -SO_4_^2-^ which can bind NH_4_^+^ cations [22,23]. Ammonia in water accumulates in the form of NH_4_^+^, which can disrupt the life of aquatic biota, so it is necessary to reduce levels of ammonia in water [24]. Ammonia levels in waters are usually less than 0.1 mg/L. If the ammonia level is more than 0.1 mg/L, the waters are toxic to some fish. High ammonia levels can contaminate organic matter from domestic, industrial, and agricultural wastes [22]. Marlina et al. [5] have reported that polyurethane synthesized from algal biomass can reduce ammonia levels in pond water. The addition of activated carbon contributes to an increase in functional groups and surface area, which are essential for removing NH_3_-N. Adsorption capacity increased rapidly after adding activated carbon to the PUM (from 187.84 to 393.43 μg/g). The results also suggest AlgPU is a suitable matrix for the immobilization of activated carbon as fillers. The PUM has demonstrated the potential use of developed algae for NH_3_-N removal [5]. The modified membrane system AF-MBMBR (sponge moving bed membrane bioreactor coupled with a pre-positioned anoxic bio-filter) was proposed to treat saline wastewater from marine culture. The results showed that the efficiency of TOC (total organic carbon) removal was very high, namely 92.8–96.2%, and the excellent TN (total nitrogen) removal efficiency reached 93.2% [21]. The application of adsorption membranes in removing ammonia has received significant attention thanks to its outstanding performance in the hybrid process, namely the adsorption and filtration approaches [20].

The synthesis of PUM from natural materials has been widely developed, as previously mentioned, however the synthesis of PUM from red seaweed of the *Gracilaria verrucosa* Greville species has not yet been reported in the literature. This study used all parts of the seaweed type *Gracilaria verrucosa* Greville, known as seaweed biomass, with the PUM produced applied to filter the ammonia solution. The Box–Behnken Design of Response Surface Methodology (RSM) was used to obtain the optimal composition in this study. RSM is a mathematical and statistical method that can be used for modeling and analysis to see the effect of quantitative variables on response variables and optimize these variables’ results. This design can also incorporate factorial and incomplete group designs [25,26]. This design uses Design Expert Software Version 10.0.3.0 with three factors and three levels. In addition, RSM is also used to model and analyze quantitative variables. The relationship between these variables can be described in equation [27,28,29]. The response used is the level of ammonia absorbed by the PUM. The relationship between factors and responses produces a 3D graph in order to determine the optimal composition.

## 2. Materials and Methods

### 2.1. Materials and Samples

The materials and samples used were an aquadest, 1,4-dioxane as a solvent, glycerin and castor oil as plasticizers, toluene diisocyanate (TDI), and NH_4_Cl. Each material used had Pro Analysis (PA) qualities from Merck (Darmstadt, Germany). Furthermore, the Gracilaria sp red seaweeds were obtained from ponds in Lamnga Village, Mesjid Raya Subdistrict, Aceh Besar Regency, Aceh Province.

### 2.2. Equipment

The equipment used includes a Fourier Transform Infra-Red (FTIR) (IR-Prestige-21, SHIMADZU), a Particle Size Analyzer (PSA) (DelsaTM Nano Beckman Coulter), a Scanning Electron Microscope (SEM) (JOEL-6510 LA), a Differential Scanning Calorimetry (DSC) (DSC-60, SHIMADZU), a Thermogravimetric Analysis (TGA) (DTG-60, SHIMADZU), and an MTS EM tensile test with ASTM D638 Plastics Tension 1229.

### 2.3. Red Seaweed Biomass Preparation

The red seaweed obtained was sorted and cleaned of impurities using tap water and dried in sunlight for five days. After drying, it was ground using a grinder and sieved using fine gauze to obtain red seaweed biomass [30]. The red seaweed biomass (RSB) was then characterized through a size analysis using PSA, and the sample was weighed, put into a cuvette, and then combined with aqua pro injection up to 2.5 mg. The cuvette was inserted into the PSA tool holder and the functional group analysis using FTIR.

### 2.4. Polyurethane Membrane Preparation

Polyurethane membrane from red seaweed biomass (PUM-RSB) was synthesized by weighing 0.2 g RSB, which was placed in a beaker. Then 5 g of 1,4-dioxane and 0.5 g of castor oil was added and homogenized for 10 min. Next, 2.5 g of TDI and 0.3 g of glycerin were added, and the mixture was heated at 60 °C for 90 min. Afterward, the dope solution was printed using a petri dish with a thickness of 0.800 ± 0.005 mm and placed in a dust-free room at room temperature for 24 h. When the membrane sheet was fully formed, it was immersed in warm, distilled water for 1–2 h and removed from the mold [31].

### 2.5. Polyurethane Membrane Characterization

The resulting PUM-RSB was further characterized to include functional group analysis using FTIR (IR-Prestige-21, SHIMADZU), samples made into KBr pellets (ratio 1:20), and recorded spectrum in the wavenumber range 4000–400 cm^−1^. Morphological analysis was conducted using SEM (JOEL—6510 LA); the sample was placed on an aluminum plate and coated with palladium gold using a vacuum. The sample was analyzed using Det.BSE and SE at a voltage of 10, 15, and 20 kV. Thermal analysis was conducted using DSC (DSC-60, SHIMADZU) and TGA (DTG-60, SHIMADZU); the observation was carried out under a nitrogen gas flow with a speed of 20 mL per minute. The sample was weighed to 10 mg and heated at a temperature of 0 to 600 °C. The mechanical properties were analyzed using MTS EM tensile test with ASTM D638 Plastics Tension of 1229.

### 2.6. Filtration Experiment

First, 70 mL of 10 ppm NH_4_Cl test solution (pH 9) was put into 17 pieces of 100 mL beaker glass, and each test solution was inserted into a filtration module with a PUM-RSB attached with a surface area of 22.051 cm^2^. Next, the filtration process was carried out with a dead-end flow system with a pressure of 20 bar for 20 min at room temperature. The ammonia levels before and after the filtration process were analyzed using a UV-Vis spectrophotometer with the Nessler method at a wavelength of 425 nm. The water flux and rejection factors of a PUM-RSB are determined using the following Equations (1) and (2).

(1) $$\mathrm{Water}\;\mathrm{Flux}\;J=\frac{V}{\mathrm{AtP}}$$

(2) $$\mathrm{Rejection}\;\mathrm{factor}\;R=\left(1-\frac{C_{2}}{C_{1}}\right)\times 100\%$$

J: Flux (mL/cm^2^·min·bar)
V: Permeate volume (mL)
A: Surface area (cm^2^)
t: Time (min)
P: Pressure (bar)
R: Rejection factor (%)
C_1_: Feed concentration (ppm)
C_2_: Permeate concentration (ppm)

## 3. Results and Discussion

### 3.1. Red Seaweed Biomass

**Figure 1:**
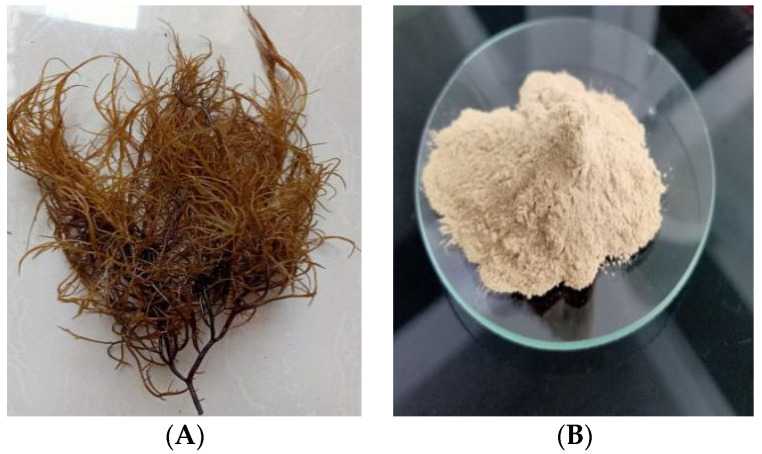
Red seaweed (*Gracilaria verucosa* Greville) (**A**) and red seaweed biomass (RSB) (**B**).

The red seaweed used in this study was from the Species: *Gracilaria verrucosa* Greville, Genus: *Gracilaria*, Familia: *Gracilariaceae*, Order: *Gracilariales*, Class: *Rhodophyceas*, which had been identified in the plant systems laboratory of the Faculty of Biology, Gadjah Mada University. The *Gracilaria verrucosa* Greville is a red seaweed (Figure 1) that grows wild in community ponds and is not utilized for many commercial purposes. The red seaweed used was obtained from Lamnga Village, Aceh, Indonesia. All parts of the red seaweed, alternatively known as biomass in the form of red seaweed biomass (RSB), were produced from the algae milling process.

The particle size is a significant factor in the reaction rate, where the smaller the particle size, the faster the reaction process [32]. Based on the PSA data, the RSB had a particle size of 777.3 nm with a polydispersity index of 0.221. The closer the polydispersity index value is to 0, the more homogeneous the particle size distribution, whereas a value above 0.5 indicates a heterogeneous particle size distribution [33].

**Figure 2:**
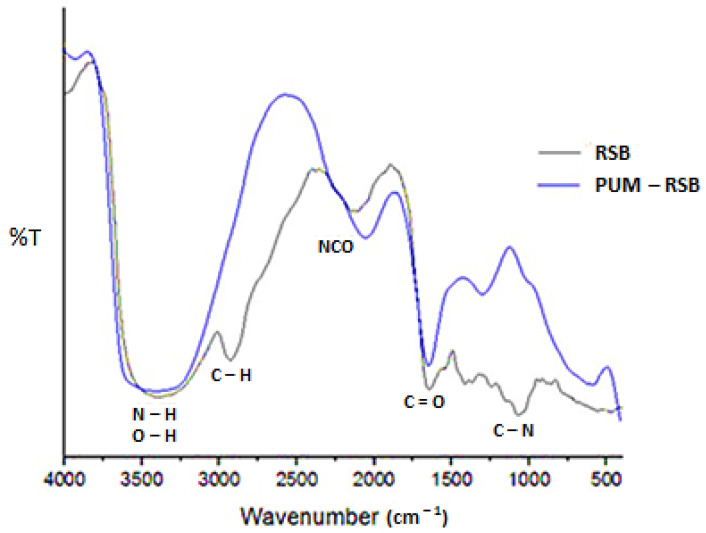
FTIR spectrum of red seaweed biomass (RSB) and polyurethane membrane from red seaweed biomass (PUM-RSB).

The FTIR spectrum of RSB can be seen in Figure 2. The spectrum showed a deep, wide absorption at 3404 cm^−1^, which signifies a large number of -NH and -OH groups which are possible candidates for forming urethane bonds [8]. In addition, the weak absorption at 2920 cm^−1^ for C-H alkanes can result in brittle and less elastic membrane properties. In addition to the abundant -OH groups, the transmittance value shows the complexity of the compounds contained in the RSB FTIR spectrum. Transmittance is defined as the ratio of light intensity before and after passing through the sample (T = I/Io). The lower the transmittance value, the more light is absorbed by the functional groups in the sample, meaning that the functional groups contained in the sample have a large number and are complex [34].

### 3.2. Polyurethane Membrane

**Figure 3:**
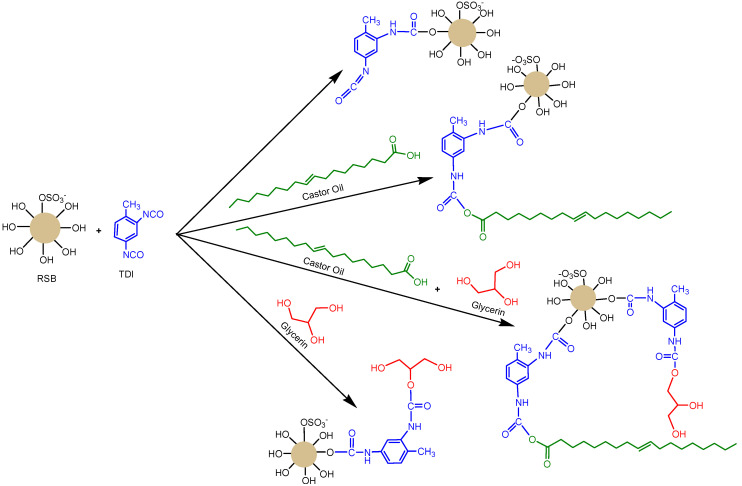
Reaction of PUM-RSB synthesis [1,36,39].

The synthesis of PUM using red seaweed biomass (PUM-RSB) was carried out by trial and error until the right conditions were ascertained. Synthesis required the formation of urethane bonds based on two main groups, namely hydroxy and isocyanate. The polymerization reactions of polyurethane formation are shown in Figure 3, where urethane cross-linkage occurred in the presence of a functional group, such as a hydroxy and isocyanate group [15]. Furthermore, the composition of its materials or ingredients significantly influences the physicochemical properties of the PUM-RSB, as it forms both soft and hard segments [35]. Its synthesis involved a variety of additional materials (Table 1).

In Table 1, it can be seen that the PUM-RSB has a brittle and inelastic nature; this is due to the lack of soft segment constituent components. The addition of castor oil and glycerin can increase the elasticity and strength of the PUM. Castor oil can form soft segments [36], while glycerin can increase the formation of cross-links (hard segments). PUM in the form of RSB and TDI possesses brittle, breakable properties. These properties are due to the lack of soft segment-forming components, which is the weak absorption at 2929 cm^−1^ C–H alkanes (Figure 2). Therefore, other additives needed to increase the elasticity and firmness of the membrane were added, such as castor oil [37] and glycerin. The PUM-RSB formed with the addition of castor oil and glycerin had dry and elastic properties. The membranes with numbers 6 and 7 are membranes without using RSB as a control.

Polyurethane membranes synthesized using 0.2 g RSB, 2.5 g TDI, 0.5 g castor oil and 0.3 g glycerin had visually shown better results (Table 1). Therefore, the membranes were characterized by methods including functional group analysis using FTIR, morphological analysis using SEM, thermal analysis using TGA and DSC, and tensile strength analysis.

The FTIR analysis of the functional groups of PUM-RSB proved that the urethane bond was formed (Figure 2). The urethane bond is characterized by the absorption of -NH bonds at 3390 and 1635 cm^−1^, -C=O at 1738 cm^−1^, -CN at 900–1300 cm^−1^, and weakening -NCO 2280 cm^−1^ [38]. The increase in absorption of -NH bonds at 3390 cm^−1^ compared to the FTIR of RSB indicates the addition of -NH bonds formed from urethane bonds and the reduction of -OH bonds from RSB.

**Figure 4:**
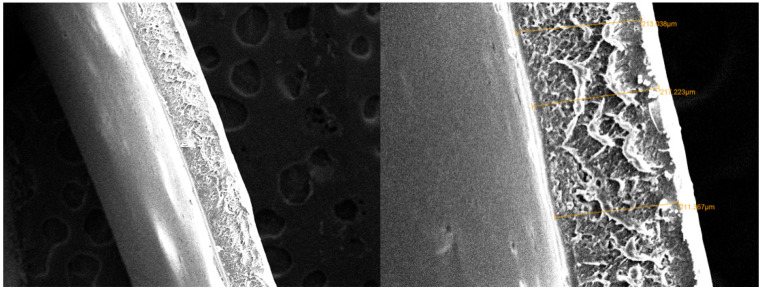
Cross-section of PUM-RSB with a magnification of 60 and 200×.

Furthermore, the SEM image in Figure 4 shows a layer formation, and the outer layer looked tighter than the inner. Therefore, the outer layer has the potential to maintain the strength of the membrane and as a selective layer. The inner layer contains a cavity, which serves as a membrane reinforcement and a filter. In the inner layer, gaps are formed due to dense cross-bonding of hollow urethane cross-linking [40,41].

**Figure 5:**
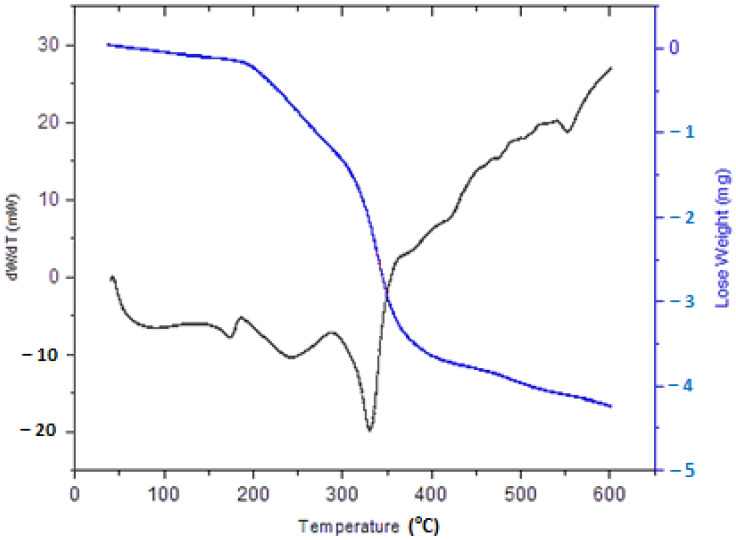
TGA and DSC curves of PUM-RSB.

Thermogravimetric Analysis (TGA) was applied against temperature. The initial degradation temperature depended on the thermal stability of the weakest point in the macromolecular structure. The second degradation temperature was dependent on the urethane bonds formed and the most thermostable units, aromatic, and ester groups of soft segments in the macromolecular structure. Meanwhile, the final degradation temperature was dependent on either the formed polyurethane bond or some other structure [36]. PUM-RSB generally has low thermal stability, as the urethane groups are unstable and decompose below 300 °C. Therefore, a reduction in the initial membrane weight at 300 °C influences the degradation of urethane and urea bonds present in the hard segment. The second thermal degradation between 340–450 °C involved further breaking of bonds into other aliphatic groups present in the membrane structure [42]. The TGA results showed that the membrane had a high initial degradation temperature of 290.71 °C with a residue of 4.88%, as demonstrated in Figure 5. In addition, the high degradation temperature occurred due to the cross-linkage of urethane [43]. Moreover, DSC analysis of the PUM-RSB showed the first endothermic peak at 95 °C was assigned to water evaporation. The urethane bond-breaking was detected at an endothermic peak at 242 °C, a high temperature indicating a more thermally stable urethane linkage. The peak in 300–500 °C is associated with some degradation of the hemicellulose complex constituents [5,6,37].

**Figure 6:**
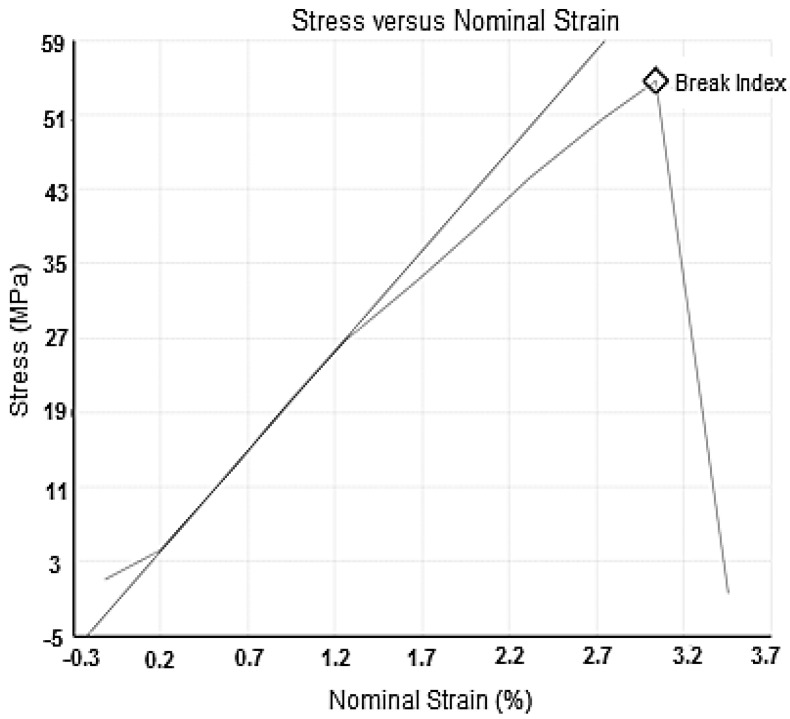
Tensile strength curve of PUM-RSB.

The mechanical properties of the resulting PUM-RSB have a stress value of 53.43 MPa and a nominal strain of 2.85%, as shown in Figure 6. From the results of the mechanical properties test, the resulting PUM-RSB is still less elastic. Membrane strength and strain significantly affect membrane performance. The PUM synthesized using carrageenan has a 9% elongation percentage. This membrane also has a large tensile strength of 340 kgf/mm^2^, with a yield strength of 69.17 kgf/mm^2^ [13]. The base material for the synthesis of PUM-RSB can affect the elongase value and tensile strength.

### 3.3. Ammonia Filtration

**Figure 7:**
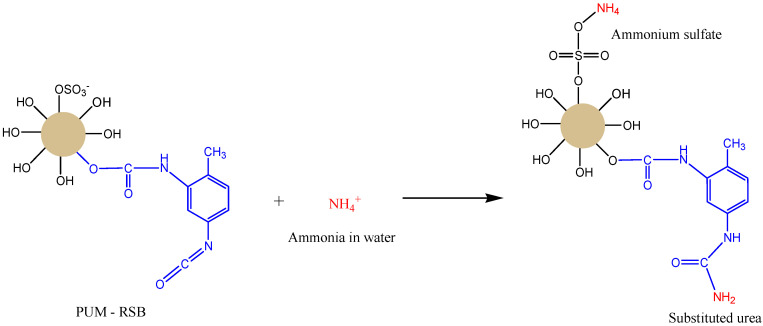
Interaction of ammonia in water by a PUM-RSB [23,24].

Polyurethane membranes synthesized using red seaweed biomass (PUM-RSB) have been applied as an ammonia filter in water. PUM-RSB can be used as an ammonia filter because PUM-RSB has a free SO_4_^2−^ anion group and an isocyanate (NCO) group. Both groups can bind NH4^+^ cations in water with the reaction shown in Figure 7. The reaction between SO_4_^2−^ anions and NH_4_^+^ cations will form ammonium sulfate bonds, while the reaction of the isocyanate group (NCO) with the NH_4_^+^ cation will form a substituted urea bond [23,24]. These bonds very rarely occur because of the limited SO_4_^2−^ anion groups and isocyanate groups (NCO) on the surface of the PUM-RSB.

The optimal PUM-RSB membrane was determined by varying the composition of the membrane components. Varied membrane composition using a combination design using Response Surface Methodology with Box–Behnken Design, which can combine factorial designs with incomplete group designs [25]. The results of this design use Software Design Expert Version 10.0.3.0 with three factors (RSB, TDI, and Glycerin) and three levels (low, medium, and high), resulting in 17 run combinations of the PUM synthesis composition as shown in Table 2.

Table 2 shows the results of decreasing ammonia levels from the filtration process using a PUM-RSB. The highest ammonia level that can be maintained by PUM-RSB is a flux of 1.660 mL/cm^2^.min.bar and a rejection factor of 32.369%. The low rejection factor is caused by the low interaction between ammonia and sulfate and isocyanate groups on the PUM-RSB. The interaction between ammonia and PUM-RSB was strongly influenced by the pH of the feed solution [44], while, in this study, pH 9 was used. At alkaline pH ammonia will be in the form of NH_3_ while at acidic pH ammonia in water will be NH_4_^+^, which allows NH_4_^+^ to bond with SO_4_^2−^ at MPU and form ammonium sulfate bonds [23,24]. The value of the rejection factor and water flux in this study was lower compared to other studies. The application of clinoptiloite-based hollow fiber ceramic membranes in ammonia ffiltration resulting in a rejection factor of 96.67% and a flux of 30 L/m^2^·h [2]. Meanwhile, another study used polyurethane films from polyol algae with activated carbon filler to remove ammonia using the adsorption method showed a high adsorption capacity of 109.45% [5]. Comparison of the results from the literature can be used for further development of this PUM-RSB research using the adsorption method and the addition of activated carbon as filler.

### 3.4. Statistical Design Model

All types of model designs show no significant results, which can be seen from the higher R^2^ value for the response of the rejection factor. The highest R^2^ value is the quadratic model reaching 57.46%, while the linear model is the linear model 14.74%, and the 2FI model 22.31%. The R^2^ value of all models has not yet produced the desired value; a reasonably good R^2^ value is above 70%. The value of R^2^ expressed in % can indicate the contribution of the regression. The greater the R^2^ value, the greater the contribution or role of factor (x) to the response (y) [25]. The rejection factor statistical design model can be seen in Table 3 and the analysis of the variance of the quadratic model in this study can be seen in Table 4.

**Figure 8:**
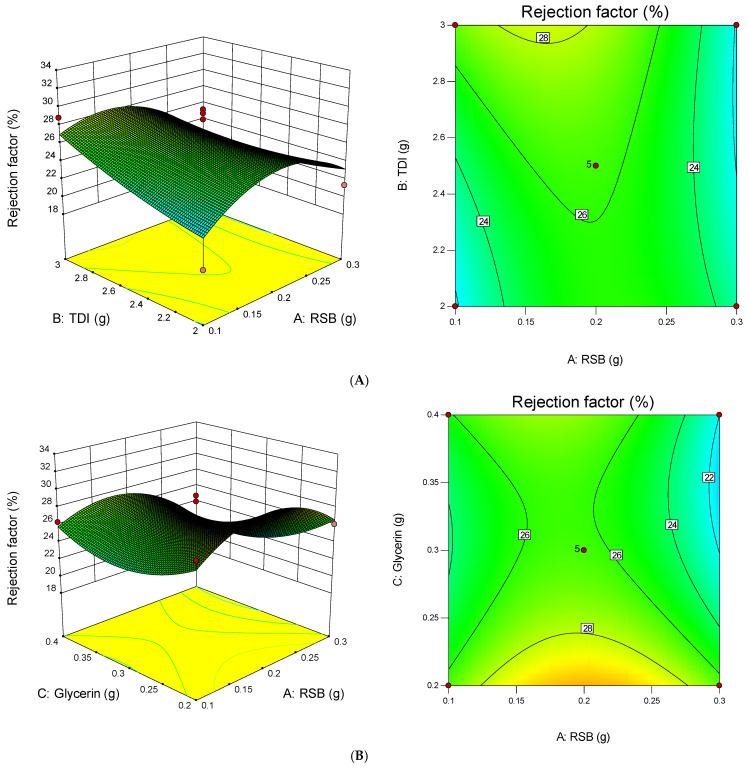

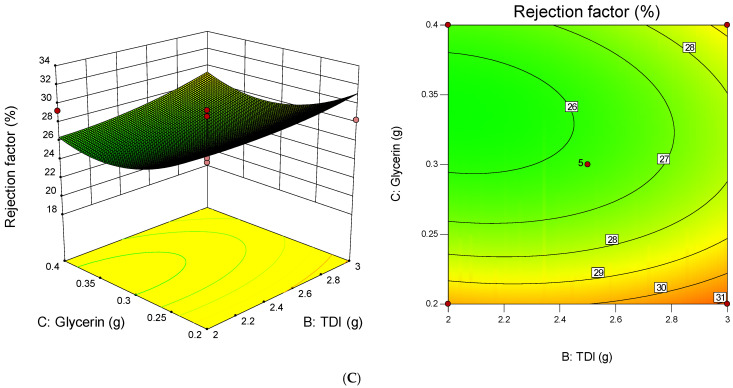
A 3D plot and surface plot of the relationship between (**A**) RSB and TDI to rejection factor, (**B**) RSB and Glycerin to rejection factor, and (**C**) TDI and Glycerin to rejection factor.

The Adeq Precision value is the signal-to-noise ratio. The expected ratio is greater than 4, in the quadratic model the resulting ratio is smaller than 4, this indicates an inadequate signal [45]. The relationship between the PUM-RSB rejection factor and the factor (x) based on the coefficient value can be seen in Equation (3) and the 3D plot in Figure 8.

y = 26.29 − 0.95A + 0.98B − 1.34C − 1.63AB − 0.96AC + 0.42BC − 3.40A^2^ + 0.58B^2^ + 2.36C^2^ (3)

The optimization results using the Response Surface Methodology with Box–Behnken Design provide a solution to the composition of the PUM-RSB, as shown in Table 5. The table shows that the theoretical rejection factor results from the optimum solution are 31.324%, with a desirability of 0.925, and the desirability value is close to 1.00 [46]. The result of the rejection factor experimentally from the optimum membrane solution was 23.9%.

## 4. Conclusions

Red seaweed from the *Gracilaria verucosa* Greville can be used as a base for making PUM-RSB. The addition of castor oil and glycerin can improve the physical properties of PUM-RSB. PUM-RSB without using plasticizer is dry, brittle, and easily crushed, so the addition of castor oil and glycerin as a blaster can improve the physical properties of PUM-RSB. The PUM-RSB produced from red seaweed possesses elastic, dry, and sturdy properties. Furthermore, it had a high initial degradation temperature of 290.71 °C, and the residue from TGA analysis was 4.88%. The quadratic model chosen in the Box–Behnken design has a higher R^2^ value than other models, namely 57.46%. The optimal composition of PUM-RSB on the Box–Behnken design on Response Surface Methodology is RSB 0.176 g, TDI 3.000 g, and glycerin 0.200 g, which yields a theoretical rejection factor of 31.3% and, experimentally, 23.9%.

## Figures and Tables

**Table 1:** Variations in PUM synthesis using red seaweed biomass.

RSB ^1^ (g)	TDI ^2^ (g)	Castor Oil (g)	Glycerin (g)	Visual Description PUM-RSB ^3^	
0.2	2.5	0	0	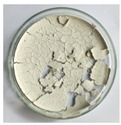	dry, brittle, crumbles easily
0.2	2.5	0.5	0	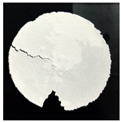	less dry, less elastic, not brittle
0.2	2.5	1	0	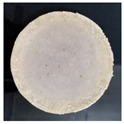	less dry, less elastic
0.2	2.5	0.5	0.3	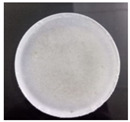	dry, elastic, and not easily torn
0.2	2.5	0	0.3	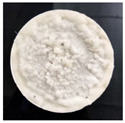	dry, stiff, less elastic
0	2.5	0.5	0.3	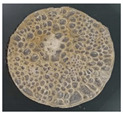	dry, hard as glass
0	2.5	0	0.3	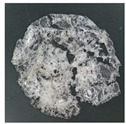	dry, hard foam

^1^ RSB: Red seaweed biomass, ^2^ TDI: Toluene diisocyanate, ^3^ PUM-RSB: Polyurethane membrane from red seaweed biomass.

**Table 2:** The result of decreased ammonia levels from the filtration process.

Run	Factor 1 A: RSB (g)	Factor 2 B: TDI (g)	Factor 3 C: Glycerin (g)	Flux (mL/cm^2^·min·bar)	Rejection Factor (%)
1	0.2	3.0	0.4	0.952	26.944
2	0.2	2.5	0.3	0.700	24.231
3	0.3	2.5	0.4	0.731	21.037
4	0.2	2.0	0.2	1.660	32.369
5	0.2	3.0	0.2	0.935	28.330
6	0.1	2.5	0.4	0.930	26.342
7	0.2	2.5	0.3	1.322	28.692
8	0.1	2.0	0.3	0.754	18.505
9	0.3	2.0	0.3	0.732	21.339
10	0.1	2.5	0.2	1.156	27.547
11	0.3	2.5	0.2	0.928	26.100
12	0.2	2.5	0.3	1.141	29.355
13	0.2	2.0	0.4	1.662	29.295
14	0.3	3.0	0.3	0.701	25.196
15	0.2	2.5	0.3	0.840	25.437
16	0.2	2.5	0.3	1.058	23.749
17	0.1	3.0	0.3	0.992	28.873

**Table 3:** Statistical design model of PUM-RSB synthesis.

Source	Linear	2FI	Quadratic
Std. Dev	3.61	3.93	3.47
R-Square	0.1474	0.2231	0.5746
Adj R-Square	−0.0494	−0.2431	0.0277
Pred R-Square	−0.6545	−2.4505	−3.8734
Adeq Precisior	2.651	2.702	3.495
PRESS	328.60	685.27	967.87

**Table 4:** ANOVA analysis for a quadratic model of the flux and rejection factor.

Source	Sum of Squares	df	Mean Square	F Value	*p*-Value Prob > F	Characterization
Model	114.12	9	12.68	1.05	0.4853	Not significant
A-RSB	7.21	1	7.21	0.60	0.4649	
B-TDI	7.67	1	7.67	0.64	0.4514	
C-Glycerin	14.39	1	14.39	1.19	0.3111	
AB	10.60	1	10.60	0.88	0.3799	
AC	3.72	1	3.72	0.31	0.5960	
BC	0.71	1	0.71	0.059	0.8150	
A^2^	48.57	1	48.57	4.02	0.0849	
B^2^	1.42	1	1.42	0.12	0.7412	
C^2^	23.45	1	23.45	1.94	0.2060	
Residual	84.48	7	12.07			
Lack of Fit	57.90	3	19.30	2.90	0.1649	Not significant
Pure Error	26.59	4	6.65			
Cor Total	198.60	16				

**Table 5:** Optimum composition solutions for polyurethane membrane synthesis.

RSB (g)	TDI (g)	Glycerin (g)	Rejection Factor (Theory) (%)	Desirability	Rejection Factor (Experiment) (%)
0.176	3.000	0.200	31.324	0.925	23.870

## Data Availability

Not applicable.
